# Retrospective study on HIV infected pregnant women and their babies in the Western part of Romania

**DOI:** 10.1186/1471-2334-13-S1-O11

**Published:** 2013-12-16

**Authors:** Voichița Lăzureanu, Teodora Moisil, Virgil Musta, Rodica Costa

**Affiliations:** 1 Dr. Victor Babeş University of Medicine and Pharmacy, Timişoara, Romania; 2 Dr. Victor Babeş Clinical Hospital of Infectious Diseases and Pneumology, Timişoara, Romania; 3 Clinical Emergency Children’s Hospital “Dr. L. Turcanu”, Timişoara, Romania

## Background

Infection of HIV from an HIV-positive mother to her child during pregnancy, labor, delivery or breastfeeding is called mother-to-child transmission (MTCT). A very important aim of combined antiretroviral therapy (cART) is that of eradicating MTCT, having healthy babies and keeping their mothers alive.

## Methods

This is a retrospective study on 104 pregnant women with HIV, between January 2001 – January 2013 in the Western part of Romania (counties: Timiş, Arad, Caraş-Severin, Hunedoara).

## Results

Out of the 104 pregnant women, 94 were known with HIV before getting pregnant, 65.2% of them were under cART by the time they got pregnant. These patients were treated with different cART, based on their antiretroviral treatment history and guidelines. The rest of pregnant women were diagnosed with HIV at delivery or soon after childbirth. We had a total of 115 children born by mothers with HIV, out of them 72 (62.6%) were seroreverters and 17 (14.78%) were children infected with HIV. The rest of 26 children are under 18 months of age and still having positive serology for HIV. All women under cART gave birth to non-infected children. 9 newborns died at delivery or shortly after; their mothers had been diagnosed with HIV during labor (or soon after) or had refused cART.

## Conclusion

In order to prevent MTCT and have healthy babies with surviving mothers, cART taken by the women before getting pregnant is an essential factor. Sustained medical (infectious diseases plus gynecologist) and psychological efforts for caesarian section at week 38, advice for no breastfeeding and administration of pediatric antiretroviral formulas to the newborn remain essential steps in the project to eradicate pediatric HIV infection until 2015.

